# Retraction: miR-599 Inhibits Vascular Smooth Muscle Cells Proliferation and Migration by Targeting TGFB2

**DOI:** 10.1371/journal.pone.0282983

**Published:** 2023-03-29

**Authors:** The PLOS ONE Editors

The *PLOS ONE* Editors retract this article [1] because it was identified as one of a series of submissions for which we have concerns about peer review, authorship, and similarities across articles. These concerns call into question the validity and provenance of the reported results. We regret that the issues were not addressed prior to the article’s publication.

BX, CZ, and SJ either did not respond directly or could not be reached. KK responded but expressed neither agreement nor disagreement with the editorial decision.
